# Supplementary material for: Characterization of RNA Sensing Pathways in Hepatoma Cell Lines and Primary Human Hepatocytes
Source: Cells. 2021 Nov 4;10(11):3019. doi: 10.3390/cells10113019 (PMC8616302; doi:10.3390/cells10113019)
Supplement: Supplementary file 1 [file cells-10-03019-s001.zip › cells-1414246-supplementary.pdf]

Supplementary Materials:

Table S1. Clinical characteristics of PHH donors.

Donor	Year of birth	Gender	Diagnosis	Figure and symbol used in bar graph
1	1969	Male	Liver metastasis	Fig.1a,2a, 3, 4, S1, S2, S3 ●
2	1975	Male	Klatskin tumor	Fig.1a,2a, 3, 4, S1, S2, S3 ■
3	1970	Male	Klatskin tumor	Fig.1a,2a, 3, 4, S1, S2, S3 ▼
4	1949	Male	Liver metastasis	Fig.1a,2a, 3, 4, S1, S2, S3 ▲
5	1962	Female	Klatskin tumor	Fig. 5 ◆
6	1958	Female	Hemangioma	Fig. 5 ⬣
7	1986	Female	Liver adenoma	Fig. 5 ★
8	1942	Female	Klatskin tumor	Fig. 5 ◒
9	2018	Male	Liver metastasis	Fig. 5 ❋

Table S2: Primer sequences of innate immune sensors and adaptors

Target gene	Accession number	Primer	Sequence (5′-3′)
GapDH	P04406	for	GAAGATGGTGATGGG
		rev	GAAGGTGAAGGTCGG
IFNAR	P17181	for	AACAGGAGCGATGAGTCTGTC
		rev	TGCGAAATGGTGTAAATGAGTCA
IFN-β	P01574	for	ATGACCAACAAGTGTCTCCTCC
		rev	GGAATCCAAGCAAGTTGTAGCTC
ISG15	P05161	for	CGCAGATCACCCAGAAGATCG
		rev	TTCGTCGCATTTGTCCACCA
MAVS	Q7Z434	for	CAGGCCGAGCCTATCATCTG
		rev	GGGCTTTGAGCTAGTTGGCA
MDA5	Q9BYX4	for	TCGAATGGGTATTCCACAGACG
		rev	GTGGCGACTGTCCTCTGAA
MxA	P20591	for	GTTTCCGAAGTGGACATCGCA
		rev	CTGCACAGGTTGTTCTCAGC
RIG-I	O95786	for	CTGGACCCTACCTACATCCTG
		rev	GGCATCCAAAAAGCCACGG
TLR3	O15455	for	TTGCCTTGTATCTACTTTTGGGG
		rev	TCAACACTGTTATGTTTGTGGGT
TLR7	Q9NYK1	for	TCCTTGGGGCTAGATGGTTTC
		rev	TCCACGATCACATGGTTCTTTG
TLR8	Q9NR97	for	ATGTTCCTTCAGTCGTCAATGC
		rev	TTGCTGCACTCTGCAATAACT
Trif	Q8IUC6	for	GCCAGCAACTTGGAAATCAGC
		rev	GGGGTCGTCACAGAGCTTG

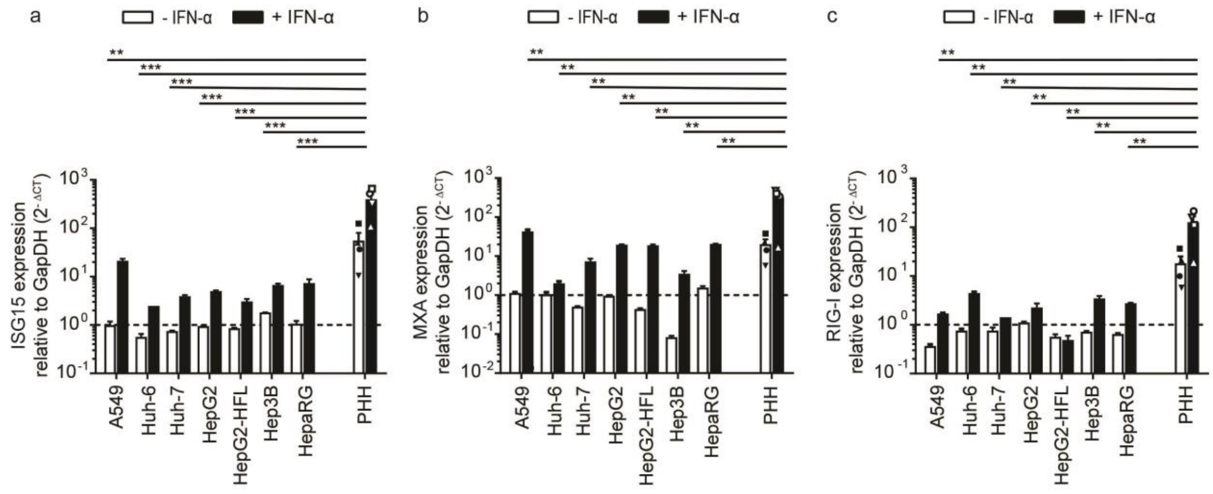

Figure S1. Induction of IFN stimulated genes in PHHs and hepatoma cell lines after IFN-α pre-stimulation and stimulation with extracellular poly I:C as a TLR3 agonist. *ISG 15* expression in PHHs and hepatoma cell lines (a), *MxA* expression in PHHs and hepatoma cell lines (b), and *RIG-I* expression in PHHs and hepatoma cell lines (c). Cells were mock treated or pretreated with IFN-α (100 IU/ml). mRNA expression was measured by qRT-PCR as described in Fig. 1a. Data for the four different PHH donors are shown as mean ±SEM of single experiments performed in technical duplicates for each donor. For the cell lines, mean ±SEM of one experiment (performed in technical duplicates) is shown. Two-way ANOVA, followed by Dunnett`s multiple comparison test ** p<0.01, *** p<0.001. Hepatoma cell lines were tested against PHH.

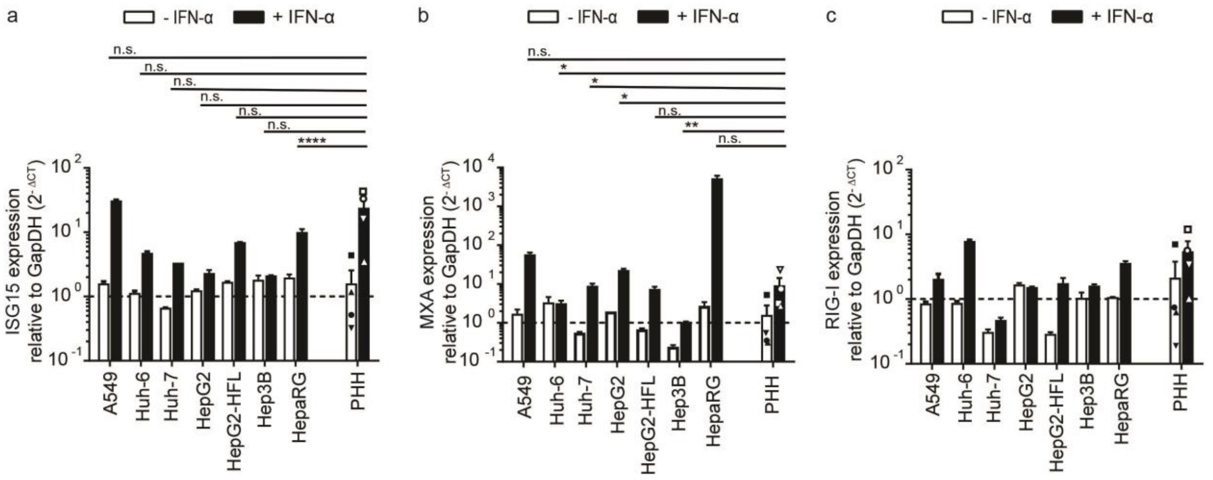

Figure S2. Induction of IFN stimulated genes in PHH and hepatoma cell lines after IFN-α pre-stimulation and stimulation with the TLR7/8 agonist R848 (1µg/ml). *ISG 15* expression in PHH and hepatoma cell lines (a), *MxA* expression in PHH and hepatoma cell lines (b), and *RIG-I* expression in PHH and hepatoma cell lines (c). Cells were mock treated or pretreated with IFN-α (100 IU/ml). mRNA expression was measured by qRT-PCR as described in Fig. 1a. Data for the four different PHH donors are shown as mean ±SEM of single experiments performed in technical duplicates for each donor. For the cell lines, mean ±SEM of one experiment (performed in technical duplicates) is shown. Two-way ANOVA, followed by Dunnett`s multiple comparison test * p<0.05, **** p<0.0001. Hepatoma cell lines were tested against PHH.

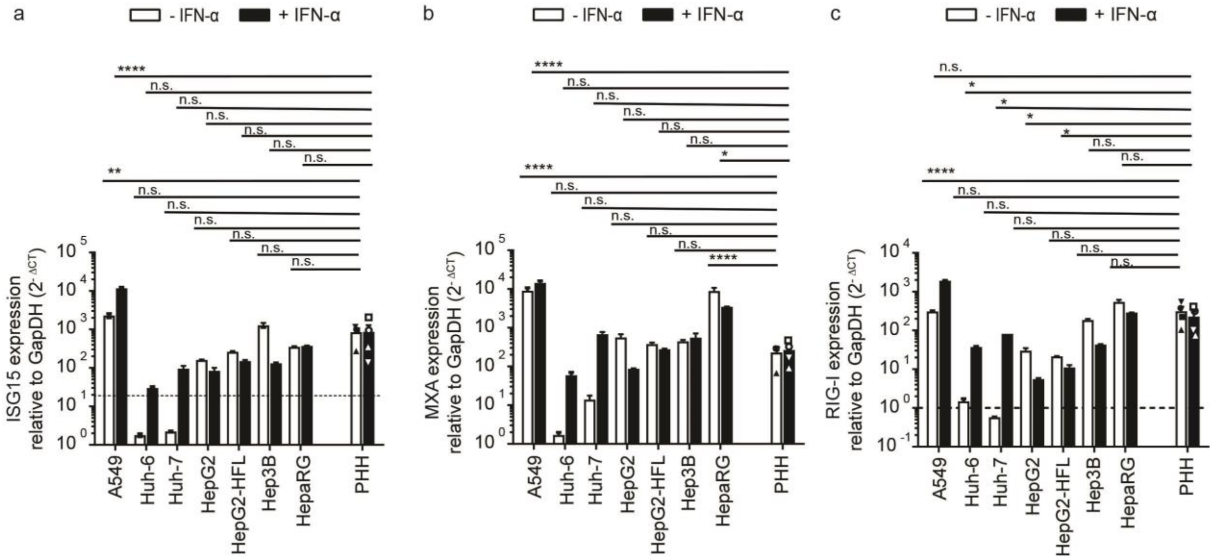

Figure S3. Induction of IFN stimulated genes in PHH and hepatoma cell lines after IFN-α pre-stimulation and stimulation with poly(I:C) (transfected, 2.25µg/well) as a RIG-I agonist. *ISG 15* expression in PHH and hepatoma cell lines (a), *MxA* expression in PHH and hepatoma cell lines (b), and *RIG-I* expression in PHH and hepatoma cell lines (c). Cells were mock treated or pretreated with IFN-α (100 IU/ml). mRNA expression was measured by qRT-PCR as described in Fig. 1a. Data for the four different PHH donors are shown as mean ±SEM of single experiments performed in technical duplicates for each donor. For the cell lines, mean ±SEM of one experiment (performed in technical duplicates) is shown.Two-way ANOVA, followed by Dunnett`s multiple comparison test * $p<0.05$, ** $p<0.01$, **** $p<0.0001$. Hepatoma cell lines were tested against PHH.
